# DNA Repair Deficiency as a Susceptibility Marker for Spontaneous Lymphoma in Golden Retriever Dogs: A Case-Control Study

**DOI:** 10.1371/journal.pone.0069192

**Published:** 2013-07-23

**Authors:** Douglas H. Thamm, Kristen K. Grunerud, Barbara J. Rose, David M. Vail, Susan M. Bailey

**Affiliations:** 1 Flint Animal Cancer Center, College of Veterinary Medicine and Biomedical Sciences, Colorado State University, Fort Collins, Colorado, United States of America; 2 Department of Medical Sciences, School of Veterinary Medicine and Carbone Cancer Center, University of Wisconsin-Madison, Madison, Wisconsin, United States of America; 3 Department of Environmental and Radiological Health Sciences, Colorado State University, Fort Collins, Colorado, United States of America; 4 University of Colorado Comprehensive Cancer Center, Aurora, Colorado, United States of America; Institute of Molecular Genetics IMG-CNR, Italy

## Abstract

There is accumulating evidence that an individual’s inability to accurately repair DNA damage in a timely fashion may in part dictate a predisposition to cancer. Dogs spontaneously develop lymphoproliferative diseases such as lymphoma, with the golden retriever (GR) breed being at especially high risk. Mechanisms underlying such breed susceptibility are largely unknown; however, studies of heritable cancer predisposition in dogs may be much more straightforward than similar studies in humans, owing to a high degree of inbreeding and more limited genetic heterogeneity. Here, we conducted a pilot study with 21 GR with lymphoma, 20 age-matched healthy GR and 20 age-matched healthy mixed-breed dogs (MBD) to evaluate DNA repair capability following exposure to either ionizing radiation (IR) or the chemical mutagen bleomycin. Inter-individual variation in DNA repair capacity was evaluated in stimulated canine lymphoctyes exposed *in vitro* utilizing the G2 chromosomal radiosensitivity assay to quantify clastogen-induced chromatid-type aberrations (gaps and breaks). Golden retrievers with lymphoma demonstrated elevated sensitivity to induction of chromosome damage following either challenge compared to either healthy GR or MBD at multiple doses and time points. Using the 75^th^ percentile of chromatid breaks per 1,000 chromosomes in the MBD population at 4 hours post 1.0 Gy IR exposure as a benchmark to compare cases and controls, GR with lymphoma were more likely than healthy GR to be classified as “sensitive” (odds ratio = 21.2, 95% confidence interval 2.3-195.8). Furthermore, our preliminary findings imply individual (rather than breed) susceptibility, and suggest that deficiencies in heritable factors related to DNA repair capabilities may be involved in the development of canine lymphoma. These studies set the stage for larger confirmatory studies, as well as candidate-based approaches to probe specific genetic susceptibility factors.

## Introduction

Cancer is largely a genetic disease associated with the accumulation of mutation and rearrangement of DNA, which results in activation of oncogenes and/or deactivation of tumor suppressor genes [1]. Although significant, the “spontaneous” rate of DNA damage accumulation is not sufficient to fully explain the high lifetime risk of cancer [2]. Thus, acquired or inherited deficiencies in the repair of DNA damage may play a key role in tumorigenesis, leading to what has been termed a “mutator phenotype” [3,4]. A variety DNA insults can produce oncogenic mutations, including ionizing radiation (IR), genotoxic chemicals, byproducts of cellular metabolism, and spontaneous DNA base damage. Additionally, there is a finite limit to the fidelity of DNA replication, which can result in occasional replication errors in the absence of exogenous or endogenous mutagens [4].

Lymphoproliferative diseases are well represented within the spectrum of human heritable cancer prone syndromes associated with defective DNA repair, for example ataxia telangiectasia (AT), AT-like syndrome, Nijmejen Breakage Syndrome, Bloom’s Syndrome and ligase IV deficiency [5]. Most lymphoma-associated heritable cancer syndromes are specifically related to deficiencies in DNA double-strand break (DSB) repair, a critical pathway for lymphocytes as they rearrange immunoglobulin or T cell receptor genes. Indeed, the most common genetic aberration in human follicular lymphoma is the t(14;18) (q32;q21) translocation, which juxtaposes the BCL2 gene with the promoter of the immunoglobulin heavy chain (IgH) gene [6]. Exclusive of the well-defined heritable cancer syndromes mentioned above, numerous studies have demonstrated enhanced mutagen sensitivity, defined generally as unrepaired DNA damage following a genotoxic stress or challenge, as a predisposing factor for human cancer [7–12]. Furthermore, family and twin studies have established that first-degree relatives of sensitive individuals are also usually sensitive, suggestive of a heritable component [13,14].

Lymphoma is one of the most common canine neoplasms [15]. The dog is an extremely useful model for the study of lymphoma in humans, owing to striking similarities in histology, biology and gene expression, including similar organ involvement, similar prognostic factors, and conserved dysregulation of signaling and growth regulation pathways [15–18]. Susceptibility to canine lymphoproliferative disease appears to be breed-related, with certain breeds developing either T-cell or B-cell neoplasia preferentially [19], again suggesting that at least some risk factors for canine lymphoma are genetically determined. Golden retrievers (GR) are markedly overrepresented for lymphoma development; a 1998 Golden Retriever Club of America National Health Survey reported a 1 in 8 lifetime risk of lymphoma in the breed [20]. Preliminary pedigree mapping of lymphoma heritability in GR suggests a possible founder effect and high coefficient of inbreeding (Jeglum KA. A descriptive pedigree of cancer in golden retrievers (abstr). In: Modiano JF, editor. Genes, Dogs and Cancer: Emerging Concepts in Molecular Diagnosis and Therapy. Keystone, CO, 2001).

Interestingly, numerous case-control studies have demonstrated that polymorphisms in DNA repair or related genes can be associated with risk of lymphomas in humans [21–27].

Here, we conducted a pilot study with 21 GR with lymphoma, 20 age-matched healthy GR and 20 age-matched healthy mixed-breed dogs (MBD) to evaluate DNA repair capability following exposure to either ionizing radiation (IR) or the chemical mutagen bleomycin. Inter-individual variation in DNA repair capacity was evaluated in stimulated canine lymphoctyes exposed *in vitro* utilizing the G2 chromosomal radiosensitivity assay to quantify chromatid-type aberrations (gaps and breaks) [28]. Sensitivity to induction of such chromosome damage in cells challenged by clastogen exposure provides an indirect measure of individual DNA repair proficiency/deficiency and cancer predisposition [29,30]. We hypothesized that: 1) lymphocytes from GR with lymphoma would display enhanced chromosomal sensitivity as compared to those from healthy GR, implying DNA repair deficiency and individual susceptibility/predisposition to lymphoma, or alternatively that; 2) lymphocytes from all GR would display enhanced chromosomal sensitivity as compared to those from healthy MBD, suggesting that GR as a breed are DNA repair deficient, and lymphoma development is a stochastic phenomenon secondary to this increased risk.

## Materials and Methods

### Ethics Statement

Informed consent was obtained from all owners, and blood collection was conducted with approval of the Institutional Animal Care and Use Committee at Colorado State University. The approval number for this protocol was 10-2007A.

### Patients and Clinical Procedures

Subjects were dogs presenting to Colorado State University, the University of Wisconsin – Madison, the University of California-Davis or Red Bank Veterinary Hospital (Tinton Falls, NJ). Twenty one GR diagnosed with lymphoma served as “cases”; none had received any specific lymphoma therapy and dogs with >1% circulating atypical lymphocytes were excluded. Twenty clinically normal, age-matched GR and 20 clinically normal, age-matched MBD served as “controls”. Lack of disease was confirmed through physical examination, owner history, complete blood count and serum biochemistry profile. Twenty mL of peripheral blood was collected into CPT^TM^ tubes (BD, Franklin Lakes, NJ) and peripheral blood mononuclear cells (PBMC) separated within 24 hours.

### Blood Processing

For each individual, multiple lymphocyte cultures were established by dividing PBMC into ten T25 flasks with 10 mL RPMI 1640 medium (Life Technologies, Grand Island, NY) containing 15% fetal bovine serum, and 50 ng/mL phorbol 12-myristate 13-acetate and 1 µg/mL ionomycin (Sigma, St. Louis, MO) to stimulate lymphocytes into the cell cycle. Cells were incubated under standard conditions (37^o^C, 5% CO_2_, humidified) for 72 hours, followed by clastogen challenge.

### Mutagen Challenge

Irradiations were performed using a J.L. Shepherd Model Mark I-68 6000 Ci ^137^Cs irradiator at a dose rate of 0.25 Gy/min. Exponentially growing canine cell cultures were irradiated with either 0.0, 1.0 or 2.0 Gy, incubated for 1, 4, or 24 h, then arrested in mitosis with 0.1 µg/mL colcemid (Sigma) for 1 h, thereby capturing cells in metaphase that had been irradiated in G2 of the cell cycle (1 and 4h) for analysis of chromatid-type aberrations (see below). Similarly, exponentially growing canine cell cultures were exposed to the radiomimetic agent bleomycin (30 µg/mL, Sigma) for 4 hours, followed by either immediate colcemid-mediated mitotic arrest, or removal of bleomycin and 20-hour incubation prior to colcemid-arrest; control cells were untreated and colcemid-arrested at identical time points.

### G2 Chromosomal Assay

The G2 chromosomal assay assesses chromatid-type aberrations (gaps and breaks) in metaphase chromosomes, which occur in cells exposed during G_2_ phase of the cell cycle [28]. Numerous human studies have utilized this methodology as a measure of chromosomal radiosensitivity and low penetrance predisposition to cancer [11,14,30,31]. Here, canine cells were processed by standard cytogenetic techniques [32]: briefly, cells were pelleted, resuspended in 5 mL 75 mM KCl and incubated for 30-minute at 37^o^C, then fixed in 500 µL 3:1 methanol: glacial acetic acid, washed 3 additional times with fresh fixative and dropped onto glass slides. Slides were stained with 5% Giemsa for 8 minutes, followed by examination with light microscopy (Zeiss Axioplan). Chromatid breaks were defined as discontinuities along the length of a chromatid with proximal and distal pieces separated by more than the width of the chromatid and were scored (blinded) in 1,000 chromosomes per condition; chromatid gaps were defined as discontinuities less than the width of a chromatid and were included in the total number of “breaks”. Individual conditions were excluded from analysis if there were <1,000 scoreable chromatids, and individual cases were omitted from analysis if >50% of all conditions had to be excluded.

### Statistical Analysis

Analyses were performed using Prism v 5.0d for Macintosh (GraphPad, La Jolla, CA). Normality of data was assessed using the D’Agostino Pearson test. Chromatid breaks over time were compared using 2-way repeated measures ANOVA followed by Tukey’s multiple comparisons analysis for each condition, comparing the three different dog groups at each evaluated time point. Sensitivity was also assessed categorically using the 75^th^ percentile of number of breaks per 1,000 chromosomes in the normal MBD as a benchmark, as established in humans [11], and “sensitive” versus “insensitive” populations compared using a 2-tailed Fisher’s exact test. Correlation between IR and bleomycin sensitivity, as well as between age and sensitivity, was assessed using linear regression. For all analyses, *p* values < 0.05 were considered significant.

## Results

The mean ages (+/- SD) of the dogs were 8.6 +/- 2.1, 8.0 +/- 2.3 and 7.25 +/- 1.9 years in the normal MBD, normal GR and lymphoma GR, respectively. No significant age differences between groups were observed. In contrast to what is generally reported in human studies utilizing the G2 chromosomal assay, where chromatid breaks are assessed per 100 nuclei, we quantified chromatid breaks per 1,000 scoreable chromosomes. This modification was necessary due to of difficulty of scoring canine chromosomes, in that there are many of them per metaphase spread (~78), all of which are small and similar in size. In addition, a few of dogs had lymphocytes poorly responsive to mitogen stimulation or other unknown factors that resulted in <1,000 scoreable chromosomes. Rather than potentially biasing results by inclusion of these cases/conditions, they were omitted from analysis (2 of 61 enrolled cases and 17 of 531 individual conditions). Two dogs were excluded based on <1,000 scoreable chromosomes in >50% of conditions (1 normal GR and 1 normal MBD). Seventeen additional individual conditions (3.2%) were similarly excluded.

**Figure 1:**
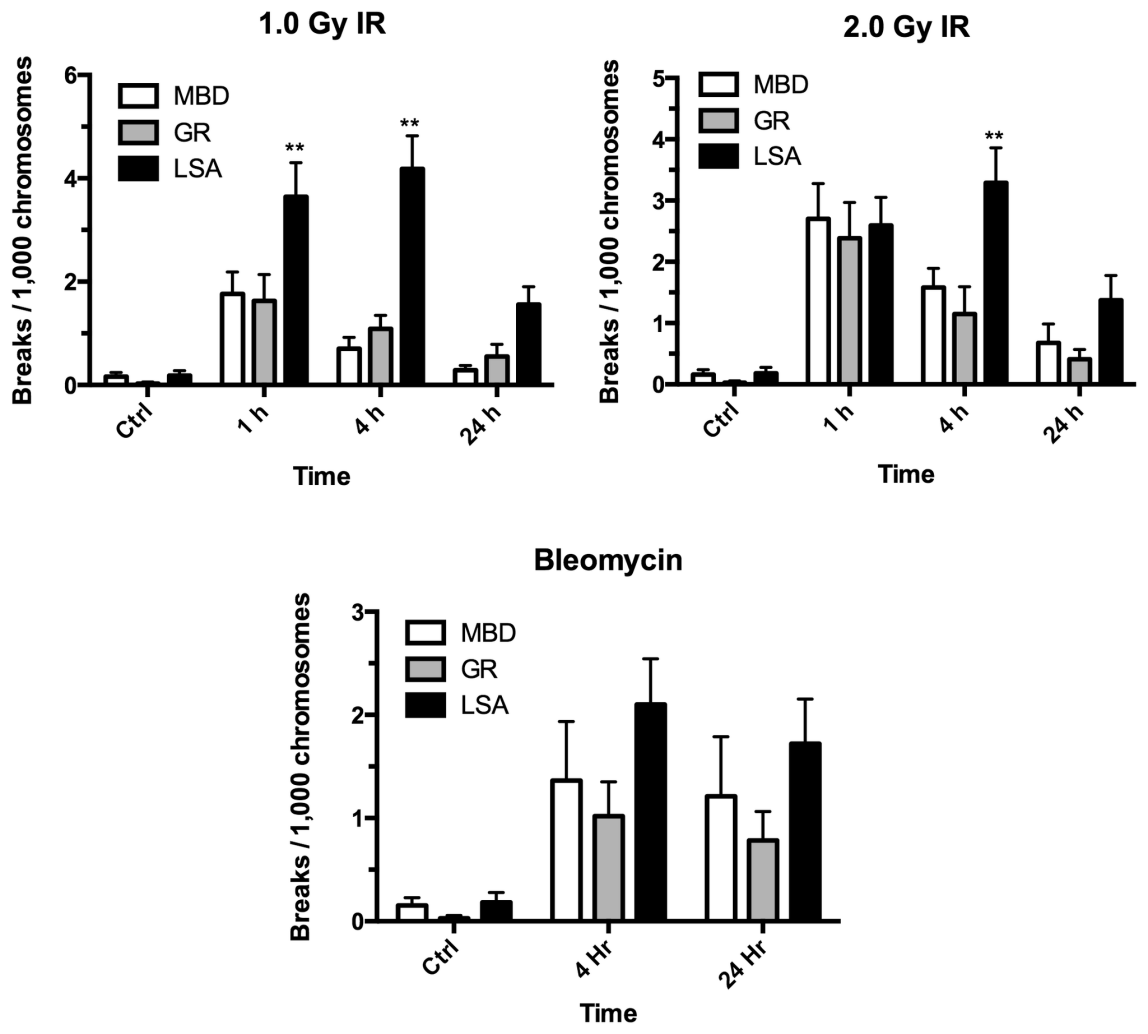
Chromatid-type aberrations in canine lymphocytes challenged with clastogen exposure. Stimulated lymphocytes (growing exponentially) from normal mixed breed dogs (MBD), healthy golden retrievers (GR) or golden retrievers with lymphoma (LSA) were exposed to 0.0 (control), 1.0 or 2.0 Gy IR, arrested in mitosis and chromatid breaks enumerated at 1, 4 and 24 hr. Spontaneous levels of chromatid breaks were not significantly different between the groups (*p* > 0.05). However, across all times following IR challenge, significantly higher frequencies of chromatid breaks were observed in golden retrievers with lymphoma compared to normal MBD or healthy GR (p < 0.0001 and 0.0089 for 1.0 Gy and 2.0 Gy respectively); increases at 24h were not statistically significant. Similar trends were observed with bleomycin treatment (p = 0.088). ** = time-points of significant difference by 2-way repeated measures ANOVA/Tukey multiple comparisons post-test. Error bars represent SEM.

No significant differences in chromatid break frequencies between normal GR and MBD under any condition were observed (Figure 1). Additionally, spontaneous (control) frequencies of chromatid breaks were not significantly different between the groups (p > 0.05). However, across all time points following IR exposure, significantly higher frequencies of induced chromatid breaks per 1,000 chromosomes were observed in GR with lymphoma compared to normal GR or MBD (*p* < 0.0001 and *p* = 0.005 for 1.0 Gy and 2.0 Gy respectively). This difference was maximal at 4 hours post-IR, consistent with exposure of cells in G2, rather than in late G2/prometaphase as could occur at 1 hr post IR. Increased frequencies of chromatid breaks at 24 h were not statistically significant, consistent with the majority of these cells being irradiated in G1, rather than in G2 (possessing chromosome-type rather chromatid-type aberrations). Similar trends across time were observed in the bleomycin-treated samples, but did not reach significance (*p* = 0.062).

**Figure 2:**
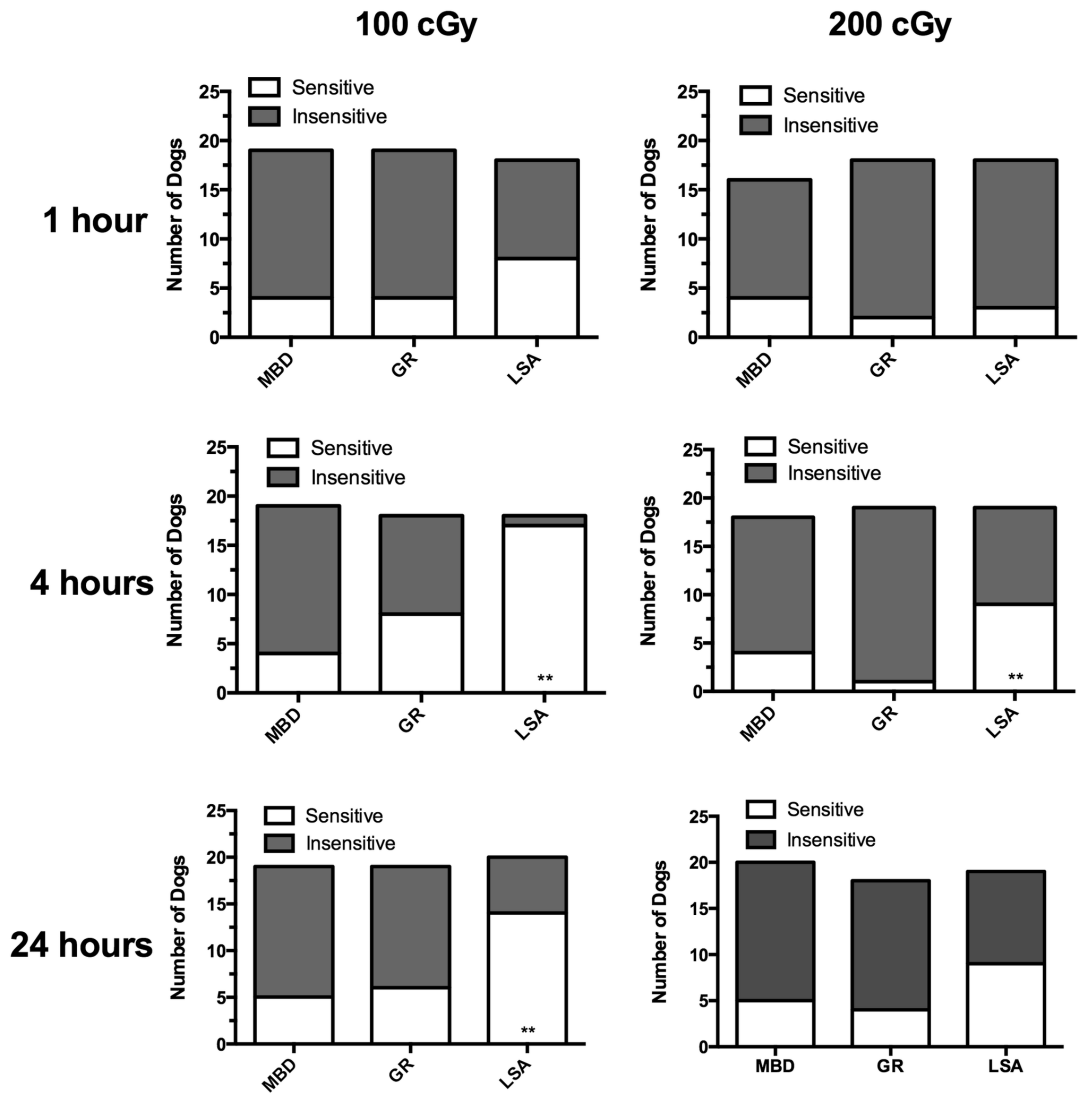
Proportions of dogs “sensitive” and “insensitive” to induction of chromosomal damage following ionizing radiation exposure. Dogs were defined as “sensitive” if the number of gross chromatid breaks exceeded the 75^th^ percentile of the reference (normal mixed-breed dog) population. The proportion of dogs demonstrating chromosomal sensitivity was significantly higher in golden retrievers with lymphoma (LSA) than in clinically normal golden retrievers (GR) at the 4-hour time point for both radiation doses and at the 24 hour time point for 1.0 cGy. A similar but insignificant trend was observed for most of the other doses and times. These results suggest compromised DNA repair capacity in the lymphoma dogs. ** = *p* < 0.05 vs. normal GR.

**Figure 3:**
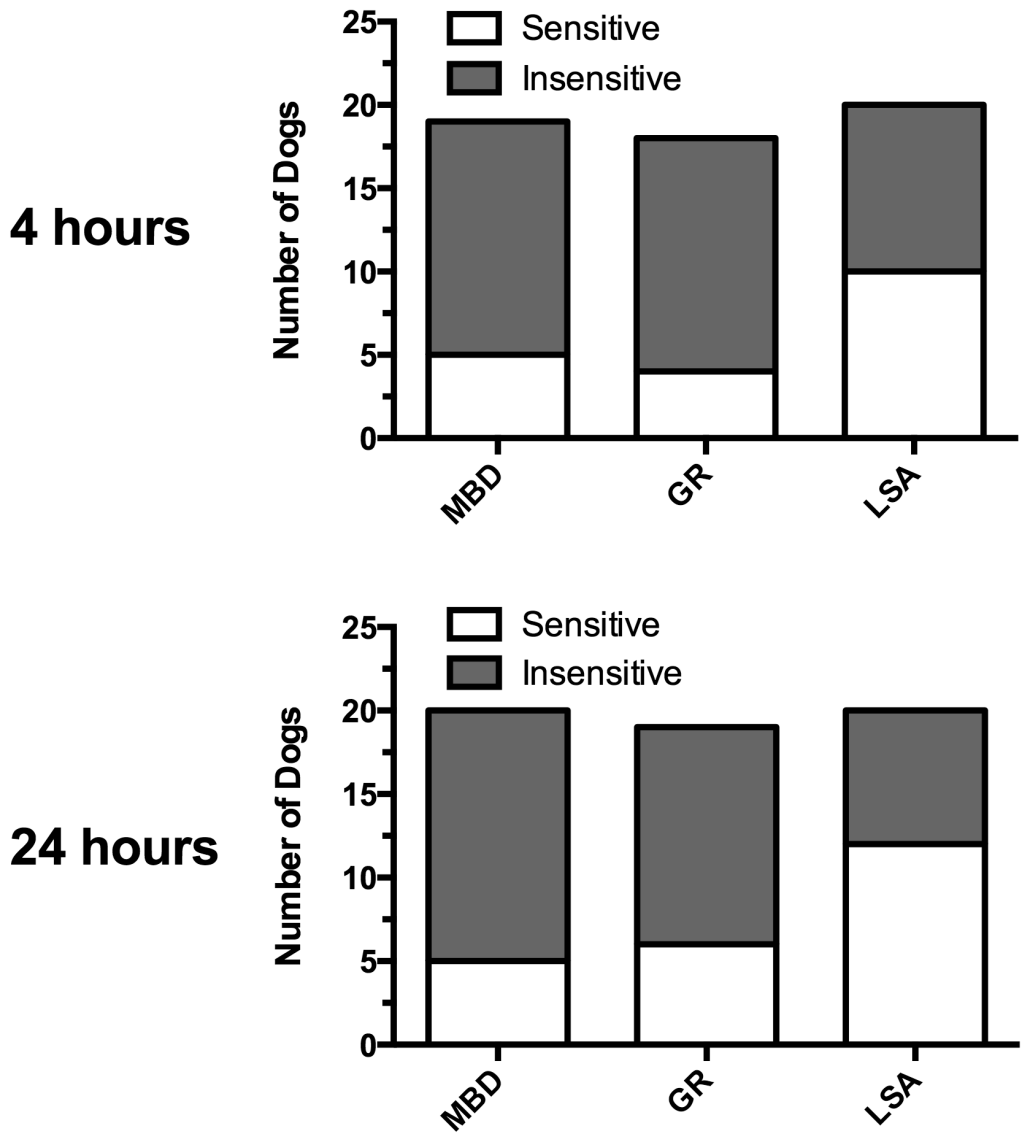
Proportions of dogs “sensitive” and “insensitive” to induction of chromosomal damage following bleomycin exposure. Dogs were defined as “sensitive” if the number of gross chromatid breaks exceeded the 75^th^ percentile of the reference (normal mixed-breed dog) population. The proportion of dogs demonstrating sensitivity to induction of damage following bleomycin exposure was higher in golden retrievers with lymphoma (LSA) than in clinically normal golden retrievers (GR) or normal mixed-breed dogs (MBD), which even though it did not reach statistical significance (*p* = 0.10 and 0.11 for 4 and 24 hours respectively, LSA vs GR), does support DNA repair deficiency in the golden retrievers with lymphoma.

The proportion of sensitive dogs (dogs exhibiting chromosome breaks in excess of the 75^th^ percentile for the normal MBD) [11] was compared between the test groups. There were no differences in the percent sensitive between normal GR and normal MBD; however, normal GR and GR with lymphoma differed significantly in three of the six IR conditions, and the trend was preserved in almost all conditions (Figures **2** and 3). Using the 4 hours post 1.0 Gy condition (the condition with maximal difference between normal GR and GR with lymphoma) to compare cases and controls, GR with lymphoma were more likely than healthy GR to be sensitive (odds ratio = 21.2, 95% confidence interval 2.3-195.8, Table 1).

**Table 1:** Diagnostic accuracy of chromosomal sensitivity (4h post 1.0 Gy) as an indication of DNA repair deficiency and marker of susceptibility to lymphoma in golden retriever dogs.

	**Normal**	**Lymphoma**
**Sensitive**	8	17
**Not Sensitive**	10	1
**Sensitivity**	0.94 (0.71-0.99)	
**Specificity**	0.55 (0.31-0.77)	
**PPV**	0.69 (0.51-0.83)	
**NPV**	0.31 (0.17-0.48)	

PPV = positive predictive value

NPV = negative predictive value

There were no significant correlations between age and number of chromatid breaks under any condition. When all dogs were combined, there were weak but statistically significant correlations between IR and bleomycin sensitivity in two of the four assessed conditions (4h post 2.0 Gy and 4h post bleomycin: r^2^ = .091, *p* = .024, 24h post 1.0 Gy and 24h post bleomycin: r^2^ = .169, *p* = 0.0014). When comparing 4h post 1.0 Gy and 4h post bleomycin (the two most discriminatory conditions between GR with lymphoma and normal GR), there was 76% concordance between classifications as sensitive and insensitive between IR and bleomycin in the normal GR (13 of 17); this was 50% (9 of 18) in the GR with lymphoma. Assessing combined sensitivity to bleomycin and IR did not significantly increase sensitivity, specificity, positive or negative predictive value.

## Discussion

Chromosomal rearrangements, including translocations, insertions and deletions are common early events in lymphomagenesis, and endogenous processes involved in antigen receptor diversification are implicated in facilitating these rearrangements [21]. Furthermore, numerous case-control studies have demonstrated that polymorphisms in DNA repair and related genes can be associated with human lymphoma risk [21,23–27]. Although there are many and assorted types of DNA damage and a variety of pathways for their repair, DSBs represent especially critical and difficult lesions to repair, which in mammalian systems are handled primarily by nonhomologous end-joining [4,5].

Spontaneous canine lymphoma can be an extremely useful model for the study of lymphoma pathogenesis, risk and therapy [15]. Genetic disease susceptibility studies in dog breeds are especially powerful, owing to dogs’ relative inbreeding and the attendant lack of genetic heterogeneity [33,34]. This power is supported by recent genetic susceptibility studies in Bernese mountain dogs and German shepherds [35,36], as well as preliminary array-CGH based studies in canine lymphoma [18]. Golden retrievers represent a breed at especially high risk of developing lymphoma in which heritability is strongly implied [20]. Here, we sought to determine whether elevated sensitivity to induction of chromosome damage in canine cells challenged by exposure to DSB-inducing agents (IR or bleomycin) could provide an indication of DNA repair deficiency in GR lymphoma, and if so, thereby identify an informative susceptibility marker. We found that lymphocytes from GR with lymphoma had significantly enhanced sensitivity to IR-induced chromatid-type breaks as compared to normal GR, suggesting that a subset of individual GR are DNA repair deficient, potentially predisposing them to lymphoma development, rather than GR as a breed being at increased risk. Assessment of chromatid breaks 4 hours post 1.0 Gy IR exposure revealed the most elevated sensitivity to induction of chromosome damage, consistent with cells irradiated in G2 phase of the cell cycle escaping a defective G2 checkpoint and entering mitosis with damage. Some cells irradiated in late G2/early prometaphase, as would be expected at the1h time point, would be in more advanced stages of chromatin condensation and therefore experience less initial chromosome breakage [37]. The G2 chromosomal assay has been used commonly in similar human studies, but it cannot distinguish between the initial DNA damage event (occurring in G2) and its repair (evaluated in metaphase chromosomes), as it simply assesses the amount of damage that persists into mitosis by enumerating chromatid-type breaks. Importantly, spontaneous levels of chromatid breaks were not different between the groups of dogs, evidence supportive of similar backgrounds in regard to initial damage. Following clastogenic challenge, however, the lymphoma GRs displayed significantly elevated levels of chromatid breaks at 1h and 4h post exposure. Based on many previous studies using this approach, as well as recent studies establishing the kinetics of DNA repair in lymphocytes [38,39], we propose that a large component of the observed sensitivity to chromosome damage reflects DNA repair deficiency in lymphoma GR.

There was only weak and inconsistent correlation between induced chromatid break frequencies following IR vs. bleomycin exposure, even though bleomycin is considered radiomimetic in that both induce DSBs, although by slightly different mechanisms. Our results most likely and simply reflect the uncertainties associated with concentration of a chemical mutagen vs. prompt and much more defined dose of IR, i.e., the number of DSBs introduced by a fixed dose of IR is relatively constant [40].

Given the fact that some dogs with lymphoma present with circulating malignant cells, we cannot rule out the possibility that occasional lymphocytes evaluated by the G2 assay represented neoplastic, rather than normal lymphocytes. To minimize this potential confounding factor, cases with >1% circulating atypical cells identified on blood smears were excluded. Thus, malignant cells should have accounted for only a very small minority of the lymphocytes scored. We also cannot rule out the possibility that some of the clinically normal GR or MBD may have been predisposed to malignancy and developed lymphoma or another neoplasm subsequent to our lymphocyte collection. We attempted to minimize this possibility by biasing enrollees toward slightly older dogs in the control groups, although the age differences between groups were not significant.

Unfortunately, it was not possible to collect pedigree information as part of the current study, so whether there is a heritable component to our observations cannot be assessed. Preliminary pedigree analysis of lymphoma in GR, however does suggest a heritable component to the disease,^a^ and it will be of great interest to determine if lymphoma risk and DNA repair deficiency cosegregate. In conclusion, the identification of a disease-associated chromosomal sensitive phenotype associated with DNA repair deficiency in GR lymphoma lays the foundation for genotype-phenotype studies and larger, confirmatory studies in lymphoproliferative disease, as well as other GR neoplasms. Furthermore, the identification of a deficiency in DNA repair, potentially of DSBs, allows for candidate-based, rather than unbiased genetic studies to interrogate specific genetic predictors of lymphoma risk in GR.
